# FISH-TAMB, a Fixation-Free mRNA Fluorescent Labeling Technique to Target Transcriptionally Active Members in Microbial Communities

**DOI:** 10.1007/s00248-021-01809-5

**Published:** 2021-08-18

**Authors:** Rachel L. Harris, Maggie C. Y. Lau Vetter, Esta van Heerden, Errol Cason, Jan-G Vermeulen, Anjali Taneja, Thomas L. Kieft, Christina J. DeCoste, Gary S. Laevsky, Tullis C. Onstott

**Affiliations:** 1 Department of Geosciences, Princeton University, Princeton, NJ 08544 USA; 2 Present Address: Department of Organismic and Evolutionary Biology, Harvard University, Cambridge, MA 02138 USA; 3 Present Address: Laboratory of Extraterrestrial Ocean Systems, Institute of Deep-sea Science and Engineering, Chinese Academy of Sciences, Sanya, 572000 Hainan China; 4 Present Address: Centre for Water Sciences and Management, North West University, Potchefstroom, South Africa; 5 Present Address: iWater Pty Ltd, 5 Walter Sisulu Rd, Park West, Bloemfontein, 9301 South Africa; 6 Department of Microbial, Biochemical and Food Biotechnology, University of the Free State, Bloemfontein, 9301 South Africa; 7 Present Address: Department of Animal-, Wildlife- and Grassland Sciences, University of the Free State, Bloemfontein, 9301 South Africa; 8 Present Address: Department of Virology, University of the Free State, Bloemfontein, 9301 South Africa; 9 Present Address: McCourt School of Public Policy, Georgetown University, Washington, DC 20057 USA; 10 Department of Biology, New Mexico Institute of Mining and Technology, Socorro, NM 87801 USA; 11 Flow Cytometry Resource Facility, Department of Molecular Biology, Princeton University, Princeton, NJ 08544 USA; 12 Confocal Imaging Facility, Department of Molecular Biology, Princeton University, Princeton, NJ 08544 USA

**Keywords:** Molecular beacons, Cell-penetrating peptides, FISH, mRNA, Methanogens, ANMEs

## Abstract

Keystone species or ecological engineers are vital to the health of an ecosystem; however, often, their low abundance or biomass present challenges for their discovery, identification, visualization and selection. We report the development of fluorescent in situ hybridization of transcript-annealing molecular beacons (FISH-TAMB), a fixation-free protocol that is applicable to archaea and bacteria. The FISH-TAMB method differs from existing FISH methods by the absence of fixatives or surfactants in buffers, the fast hybridization time of as short as 15 min at target cells’ growth temperature, and the omission of washing steps. Polyarginine cell-penetrating peptides are employed to deliver molecular beacons (MBs) across prokaryotic cell walls and membranes, fluorescently labeling cells when MBs hybridize to target mRNA sequences. Here, the detailed protocol of the preparation and application of FISH-TAMB is presented. To demonstrate FISH-TAMB’s ability to label intracellular mRNA targets, differentiate transcriptional states, detect active and rare taxa, and keep cell viability, labeling experiments were performed that targeted the messenger RNA (mRNA) of methyl-coenzyme M reductase A (*mcr*A) expressed in (1) *Escherichia coli* containing a plasmid with a partial *mcr*A gene of the methanogen *Methanosarcina barkeri* (*E. coli mcr*A^+^); (2) *M. barkeri*; and (3) an anaerobic methanotrophic (ANME) enrichment from a deep continental borehole. Although FISH-TAMB was initially envisioned for mRNA of any functional gene of interest without a requirement of prior knowledge of 16S ribosomal RNA (rRNA)-based taxonomy, FISH-TAMB has the potential for multiplexing and going beyond mRNA and thus is a versatile addition to the molecular ecologist’s toolkit, with potentially widespread application in the field of environmental microbiology.

**Supplementary Information:**

The online version contains supplementary material available at 10.1007/s00248-021-01809-5.

## Introduction

Microbial keystone species have been proposed based mostly on network analysis, and only a few of them are associated with experimental evidence [1]. Although the various definitions of “keystone species” have not been unified, some taxa have been recognized to be playing a pivotal role in shaping the structure and dynamics of their ecosystems despite their small population sizes at a particular point in time or space [1, 2]. The study of the deep biosphere, where primary productivity is basically fueled by geo-gases and inorganics, has been focused on the aspects of biosafety [3], evolution and adaptation under extreme conditions [4], and astrobiology [5]. As knowledge of the deep biosphere, such as the underexplored rare biosphere [6], and the uncharted biodiversity [7],﻿ begins to unfold, it is believed that rare, uncultured taxa could be the keystone species or ecological engineers in the deep biosphere [8]. The first step towards identifying a rare, uncultured taxon as a key or keystone species in the deep biosphere and other ecosystems is to demonstrate that the low-abundance population is metabolically active, then to characterize their genomes and physiologies, and subsequently to evaluate or perhaps even quantify their ecological importance.

To pull out information about metabolically active, low-abundance populations from that of the entire microbial community, several methods can be used. Shotgun sequencing of total RNA from environmental samples, metatranscriptomics, reveals the in situ metabolic active members in the bulk samples [8]. Then, bioinformatics analyses aid the identification of functional genes expressed by active members, providing a context to infer how rare microorganisms may interact with the rest of the community. However, the information originating from these rare members may each only account for less than 1% of the data, and it often begs for more sequencing to be performed to address the low coverage. Metatranscriptomics of total RNA is therefore not a cost-effective way for studying ecologically important members that occur at a low abundance in environmental samples. Also, analyzing and recruiting RNA information of rare taxa from metatranscriptomics datasets are deemed to be challenging in terms of bioinformatics.

Traditional fluorescence in situ hybridization (FISH) using linear probes that target the 16S ribosomal RNA (rRNA) [9] and/or messenger RNA (mRNA) [10] allow transcriptionally active cells to be visualized by microscopy. The probes provide some level of information about the taxonomic group and function of the fluorescence-labeled cells depending on the universality of the designed probe. The challenge to visualize cells with low transcript content can be alleviated by replacing linear probes with molecular beacons that have low background signal [11], and by the use of catalyze reporter deposition (CARD) [12] or hybridization chain reaction (HCR) [13] that amplifies the fluorescence signal from the target molecules. These modified FISH methods increase the target-to-background signal ratio. Combining FISH with flow cytometry and chip-based microarray meets the need for high-throughput detection and quantification, whereas combining FISH with cell sorting allows cell enrichment and separation for subsequent culture-independent work [14]. Similarly, translationally active cells from environmental samples can also be studied by employing bioorthogonal noncanonical amino acid tagging (BONCAT) methodology [15]. These labelling approaches, however, typically involve a fixation step that crosslinks proteins and nucleic acids [16], thereby inactivating the microbial cells, rendering impossible the capture of labeled rare taxa for live-cell imaging experiments and cultivation-based research.

Fixation-free 16S rRNA FISH has been applied on environmental samples and demonstrated that the two-stage sort approach enriched the target populations from 2 to 3% to 94 to 98% [17], but cultivation of labeled cells was not attempted. This in-solution fixation-free protocol involves an incubation of cells in a hybridization buffer containing 0.01% SDS and 0.9 M NaCl [17, 18] at 46 °C for 2–3 h, followed by an incubation in a wash buffer (0.9 M NaCl) at 48 °C for 20 min. The hybridization conditions, in particular the elevated temperature (presumably higher than the native temperature of the bioreactor samples, which was not stated in Ref. 18 or references therein), may stress the target cells and reduce their viability. It has not yet been tested whether this fixation-free protocol alters the physiology and viability of the target cells or not. Fixation-free protocols that employed ethanol dehydration [19] and heat shock [20] would lower the survivability of the cells, as the latter study targeting the 16S rRNA gene reported less than 3% survival rate of pure cultures. Therefore, a FISH sample preparation protocol that works under conditions similar to the native conditions of target cells will be advantageous for in situ monitoring of cellular activity and for investigating the physiology of uncultured, non-model organisms in a mixed community, which has the potential to meet the growing need to enrich, isolate, and characterize the physiology of uncultured species.

In this article, we describe the development of fluorescent in situ hybridization of transcript-annealing molecular beacons (FISH-TAMB) to label intracellular mRNA targets in prokaryotic cells. The FISH-TAMB method differs from existing FISH methodologies by the absence of fixatives or surfactants in buffers, a fast hybridization time of as short as 15 min at target cells’ growth temperature and the omission of washing steps. The initial development of the FISH-TAMB method targets the marker gene of methanogens and the uncultivated anaerobic methanotrophic archaea (ANMEs) [21], which are known to account for ~ 1–2% of read abundance in several continental subsurface microbial communities [22] and are proved to take part in the subsurface methane cycle [8, 23]. Labeling of intracellular *mcr*A transcripts, encoding for the alpha subunit of methyl-coenzyme M reductase that mediates, respectively, the last and first step of methanogenesis and anaerobic methanotrophy, was demonstrated in cells from three scenarios: (i) *Escherichia coli* cells carrying a plasmid with an insert of partial *mcr*A gene derived from *Methanosarcina barkeri* (*E. coli mcr*A^+^), (ii) an *M. barkeri* axenic culture, and (iii) ANMEs enriched from Precambrian shield subsurface fracture fluid (BE326 BH2-Conc) [8, 23]. Viability of FISH-TAMB-treated *E. coli mcr*A^+^ and *M. barkeri* cells was evaluated.

## Concept of FISH-TAMB

**Fig. 1:**
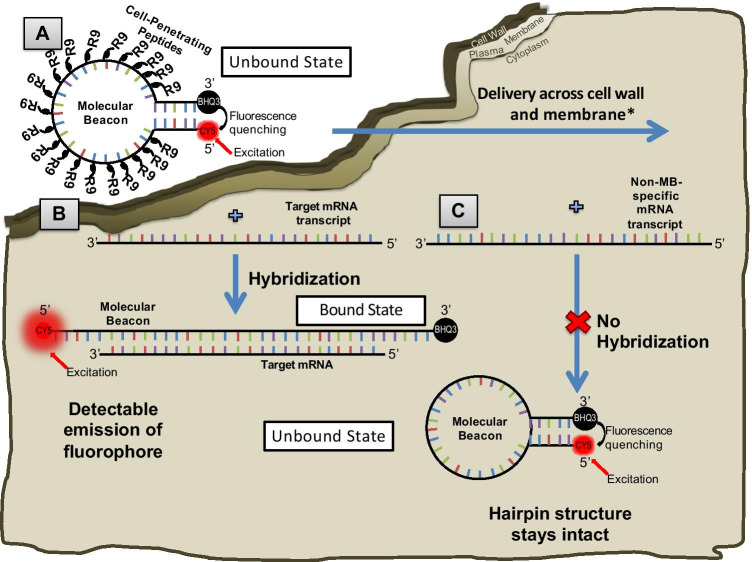
FISH-TAMB probe conformation and hybridization to encountered messenger RNAs. **A** An oligomer comprised of a 24 base-long complementary *mcr*A mRNA sequence is flanked by 5 reverse complement nucleotides to form a molecular beacon (MB) loop and stem structure. Cell-penetrating peptides (CPPs) comprising 9 arginine sequences (R9) are non-covalently bound to the MB sequence and are responsible for its delivery across the cell wall and plasma membrane. **B** Fluorescence of Cy5 fluorophore covalently bound to the 5′ end of the MB sequence remains quenched by BHQ3 bound to the 3′ terminus until the MB hybridizes to a target transcript sequence. Hybridization results in the linearization of the MB, subsequently unquenching Cy5 from BHQ3, allowing the fluorophore’s emission upon excitation by a source in the red bandwidth of the visible light spectrum. **C** If the MB encounters an mRNA transcript that is not its intended target, it will retain its hairpin conformation, and fluorescence of Cy5 will remain quenched by BHQ3. Images not to scale

Molecular beacons (MBs), with a hairpin oligonucleotide sequence outfitted with a fluorophore and a fluorescence quencher [24], are selected to target the mRNA of bacteria and archaea, as they result in a higher signal-to-background noise ratio than linear probes and have also been successfully applied to detect intracellular mRNA of living eukaryotic cells [11, 25, 26]. In the unbound state, complementary bases on the 5′ and 3′ ends of MBs self-anneal to form a stem structure, which results in fluorescence quenching. Recognition of a target sequence results in MB linearization for subsequent hybridization (Fig. 1). Thus, the fluorophore is no longer in physical proximity to the quencher, resulting in emission of a known wavelength at a level differentiable from the background fluorescence due to autofluorescence and unbound MBs.

In order to deliver the MBs into prokaryotic cells without causing cell death, cell-penetrating peptides (CPP) are used, as they have been shown to successfully deliver cargos such as DNA and nanoparticles into living cyanobacteria with negligible toxicity [27, 28]. Following the formation of FISH-TAMB probes (i.e., CPP/MB complexes) via non-covalent hybridization of MBs to CPP, FISH-TAMB probes are incubated with microbial cells in a mineral salt solution at the cells’ growth temperature. The MBs are believed to be taken up by prokaryotic cells via classical endocytosis and/or micropinocytosis [29], though it is uncertain how broadly applicable these mechanisms may be. Little is known about trafficking of the internalized FISH-TAMB probes within the cells. It is anticipated that the MBs will be dissociated from CPP due to some enzymatic reaction and then the released MBs encountering RNA molecules with a sequence complementary to the MB sequence will result in hybridization and fluorescence emission.

Cells labeled by the FISH-TAMB method can be visualized under epifluorescence and confocal microscopy, enumerated and sorted with flow cytometry and microfluidics, and collected for culture-independent and culture-dependent investigations.

## Materials and Methods

### Step 1: MB Design and Acquisition

MB sequences can be designed for a spectrum of specificity/universality: as a specific probe targeting one single taxon, as a universal probe that potentially hybridizes to all RNA variants of the target functional gene, or anywhere in between. In this study, two types of MBs were used and each comprised a GC-rich stem and 24-mer nucleotide probe sequence. The *lac*Zα MB sequence (5′-CCTGGCACTAGTGATATCGAATTCCCGCGCCAGG-3′; Integrated DNA Technologies, Inc., Coralville, IA, USA) was designed to target the region of *lac*Zα gene on the pGEM®-T Easy Vector where the insertion site is located, such that disruption of this annealing site due to gene insertion will result in no FISH-TAMB hybridization with the expressed mRNA. The *mcr*A MB sequence (5′-CCTGGCGTTCATBGCGTAGTTVGGRTAGTCCAGG-3′; Integrated DNA Technologies, Inc., Coralville, IA, USA) was modified from the reverse primer that has been commonly used in diversity studies of methanogens and ANMEs belonging to the phylum *Euryarchaeota* [30], anticipating to capture diverse cells expressing different *mcr*A genes. The underline regions are predicted to form the stem portion. Both MBs have a similar melting temperature and GC content. MBs are flanked on the 5′ end by a covalently bound Cy3 (excitation peak at 554 nm, and emission peak at 566 nm) or Cy5 fluorophore (excitation peak at 649 nm, and emission peak at 665 nm) and on the 3′ end by a Black Hole Quencher® BHQ3.

As the additional bases for the stem structure may affect the desired specificity of the MB to target mRNA, a BLAST (https://blast.ncbi.nlm.nih.gov/Blast.cgi) search against the nucleotide database was done to confirm that the MB sequences remained highly similar to the target gene sequences of target taxa. As the *lac*Zα MB sequence exactly targets a vector sequence and the *mcr*A MB sequence is derived from a well-known primer sequence, non-specific hybridization was not a concern in this study. For a newly designed probe targeting a non-laboratory-use-vector, a more vigorous evaluation of its specificity is recommended (e.g. [31]).

To minimize photodegradation, all laboratory work involving MBs was done in dim light. Solutions containing MBs and FISH-TAMB probes were kept in the dark unless otherwise specified.

### Step 2: MB Validation (Optional but Recommended)

For one who is setting up the laboratory for performing FISH-TAMB or is going to use newly designed FISH-TAMB probes, it would be a good practice to obtain empirical data about the MBs. Conducting a thorough study to characterize the thermodynamic and kinetic properties [32] of the MBs is deemed unnecessary for every application; nonetheless, it is advantageous for the user to at least find out how easily the GC stem structure may open in the absence of the target molecules, and given the detection systems available, the fluorescence levels of the unbound and bound states.

We performed two cell-free or in vitro hybridization assays on the *mcr*A MBs with a Cy3 fluorophore. Melting curve analysis was done to examine the temperature and salt effects on the integrity of the *mcr*A MBs in the presence and absence of its target sequences. Reactions of 50 μL containing 20 pmol of *mcr*A MB and 40 pmol of perfect-match target oligonucleotide (5′-ACTAYCCBAACTACGCVATGAACG-3′; Integrated DNA Technologies, Inc., Coralville, IA, USA) were prepared in sterile water and a 20-mM Tris–HCl buffer solution containing 1, 2.5, and 5 mM MgCl_2_. A set of no-template controls containing no target oligonucleotide was included. Reaction mixtures were incubated at 37 °C for an hour on a real-time qPCR 7900HT system (Applied Biosystems, Inc., Carlsbad, CA USA), followed by melting curve analysis for temperatures going from 95 to 25 °C with fluorescence signals measured every 0.2 °C at 570 nm (pre-set for NED dye). The thermal profile and data acquisition was set up using software SDS v2.3. The results (Fig. S1; Table S2) showed that the hybridization of *mcr*A MBs and target oligonucleotides (i.e., bound state) at below 65 °C and in the presence of MgCl_2_ resulted in about 27- to 193-fold increase in fluorescence intensity than the unbound state; salt is essential for keeping the stem structure, but the formation of MB-target complexes at higher concentration of salt (5 mM MgCl_2_) resulted in a lower fluorescence intensity when compared to that at lower salt concentrations, suggesting that a higher salt concentration may have increased the activation energy required for the linearization of MBs; complete dissociation of MB-target complexes occurred at ~ 65 °C and MBs were denatured to a random coil state at higher temperatures, suggesting that *mcr*A MBs may be limited from in situ studies of thermophilic methanogens and ANMEs.

A similar experiment was done using 2 pmol of *mcr*A MBs and 4 pmol of target oligonucleotides, which is 10 times less than the abovementioned experiment. The fluorescence intensities of MBs at﻿ the bound state and unbound state were low, and their differences were relatively small (at most fivefold). This degree of change may be approaching the real-time qPCR 7900HT system’s empirical detection limit for differentiating bound and unbound MBs with a Cy3 fluorophore given the hybridization condition (37 °C for an hour).

As one of the later experiments would apply FISH-TAMB for a long period of observation (> 240 min), a time-series experiment was set up to investigate the fluorescence stability of *mcr*A MBs with a Cy5 fluorophore. Reactions of 100 μL containing 40 pmol of *mcr*A MB, with or without 40 pmol *mcr*A target oligonucleotide, were prepared in 1X Dulbecco’s PBS (DPBS) solution. After incubation at room temperature for 10, 15, 20, 30, 40, 60, 80, and 100 min, the reaction mixtures were transferred to a Corning™ 96-well special optics low fluorescence assay plates (Thermo Fisher Scientific, Waltham, MA, USA). Fluorescence images were taken using a Typhoon 9410 Variable Mode Imager® (Molecular Dynamics, GE Healthcare, Little Chalfont, UK) with excitation at 633 nm, detection at 670/10 nm, and an exposure time of 5 min per image. The results (Fig. S2) showed that the fluorescence intensities of MB-target complexes and unbound MBs remained constant over the tested duration. As shown, validation experiments can be designed with conditions that are relevant to the downstream analyses, and a variety of instruments can be used for fluorescence detection.

### Step 3: Formation of FISH-TAMB Probes

CPP nine arginine residues (R9) was selected as a carrier to deliver MBs across cell walls and plasma membranes, as it has been demonstrated to penetrate cyanobacterial walls and membranes without harmful effects [27, 28]. R9, purified by high-pressure liquid chromatography, was ordered from Millipore Sigma, St. Louis, MO, USA. FISH-TAMB probes were formed by mixing R9 and MB molecules together at an optimized ratio, which can be determined by gel retardation assay.

We used a protocol modified from Liu et al. [28]. Briefly, R9 and MB stock and working solutions were prepared in 1X TE buffer (10 mM Tris–HCl, 1 mM EDTA-Na_2_, pH 8.0). Aliquots of 200 μM R9 and 10 μM MB were mixed gently in 1 × DPBS solution at varying molar ratios of R9 and MB (0:1, 5:1, 10:1, 15:1, 20:1, 25:1, 30:1). The mixtures were incubated for 30 min at 37 °C on a C1000 Touch™ Thermal Cycler (Bio-Rad Laboratories, Inc., Irvine, CA, USA) to allow for the complexation of all free-floating MBs in solution. Afterward, the mixtures were mixed with 1X DNA loading dye (Thermo Fisher Scientific, Waltham, MA USA) and analyzed by electrophoresis on a 1% (w/v) agarose gel containing ethidium bromide in 1X TAE buffer solution (40 mM Tris, pH 7.6; 20 mM acetic acid, 1 mM EDTA-Na_2_) for 30 min at 100 V. A 100 bp ladder (New England BioLabs®, Ipswich, MA USA) was used as a size marker.

For the probes used in this study, a R9 to MB molar ratio of 20:1 was found to be adequate for R9s to scavenge all free MBs in the solution (Fig. S3). Therefore, the FISH-TAMB probe working solution used in this study was prepared at a 20:1 R9:MB molar ratio (i.e., containing 20 μM R9 and 1 μM MB) using the protocol mentioned above and stored in the dark at -20 °C until use.

### Step 4: Validation of the Delivery of FISH-TAMB Probes and Subsequent Hybridization with Target Transcripts (Optional but Recommended)

Before conducting a full experiment to answer the scientific questions of interest, it is advantageous to do a procedural check to verify that the prepared FISH-TAMB probes can pass the cell membrane barrier and enter into the cytoplasm, that the internalized MBs can bind to the target molecule if present, and that the resultant fluorescence level can be confidently differentiable from the background signals. As FISH-TAMB probes are intended to be used to label intracellular mRNA under conditions similar to that of the sample’s native conditions (e.g., temperature, salinity or osmolarity, pH), it is recommended to do this in vivo validation step with conditions consistent with the future experiments.

As a proof of concept, our FISH-TAMB probes appended with Cy5 fluorophore were applied to label mRNA expressed by bacterial and archaeal cells, represented by pure cultures of *E. coli* and *M. barkeri*, as well as an ANME enrichment. The construction of *E. coli* cells with a plasmid containing a partial *pmo*A (particulate methane monooxygenase beta subunit) (pGEM-T::*pmo*A) or *mcr*A gene (pGEM-T::*mcr*A) and the cultivation conditions of all types of cells are detailed in the Supplementary Materials and Methods. These *E. coli* cells will be referred as *E. coli pmo*A^+^ and *E. coli mcr*A^+^. In this study, considering that the FISH-TAMB-labeled samples were analyzed by flow cytometry, it was required to suspend the cells in 1 × DPBS solution to reduce background particle counts.

Cells were harvested during their exponential phase with cell concentration determined via optical density at 600 nm (OD_600_) for *E. coli* using a Beckman DU® 530 Life Science UV/Vis Spectrophotometer (Beckman Coulter®, Indianapolis, IN USA) and at 550 nm (OD_550_) for *M. barkeri* [33] and ANMEs using a Hach DR/2010 Spectrophotometer (Hach Company, Loveland, CO, USA). An appropriate volume of cells, usually less than 1 μL, was added to 100 μL of 1 μM *mcr*A FISH-TAMB probe working solution (equivalent to 100 pmol MBs), to dilute the cells to ~ 10^6^ cells/mL. Cell-free reactions of 100 μL volume were set up as follows: (A) MB-only control, 1 × DPBS containing 40 pmol *mcr*A MB; (B) MB + non-specific target control, 1 × DPBS containing 40 pmol *mcr*A MB and 40 pmol *pmo*A oligonucleotide (5′-GAAYSCNGARAAGAACGM-3′; Integrated DNA Technologies, Inc., Coralville, IA, USA) modified from Luesken et al. [34]; (C) MB + specific target control, 1 × DPBS containing 40 pmol *mcr*A MB and 40 pmol *mcr*A oligonucleotide; (D) FISH-TAMB-only control, 1 × DPBS containing 100 pmol *mcr*A MB in the form of FISH-TAMB probe; and (E) FISH-TAMB + specific target control: 1 × DPBS containing 100 pmol *mcr*A MB in the form of FISH-TAMB probe and 100 pmol *mcr*A oligonucleotide. After incubation on a thermal cycler at 37 °C for 30 min, the reaction mixtures were transferred to a Corning™ 96-well special optics low fluorescence assay plates (Thermo Fisher Scientific, Waltham, MA, USA). Fluorescence images were taken using a Typhoon 9410 Variable Mode Imager® (Molecular Dynamics, GE Healthcare, Little Chalfont, UK) with excitation at 633 nm, detection at 670/10 nm, and exposure time of 5 min. The results showed that the fluorescence of unbound *mcr*A MBs in the absence of specific targets (Fig. S4A and S4B) was clearly lower than that of (mcrA MB)-(mcrA targets) (Fig. S4C), whereas the *mcr*A FISH-TAMB probes emitted undetectable fluorescence in the absence or presence of targets (Fig. S4D and S4E, respectively). In comparison, *M. barkeri*, the ANME enrichment, and *E. coli mcr*A^+^ that are known and expected to express *mcr*A mRNA yielded fluorescence signals (Fig. S4F, S4G, and S4H, respectively), indicating that the *mcr*A FISH-TAMB probes were successfully delivered into the cells. We hypothesized that after FISH-TAMB are internalized by the *M. barkeri*, the ANME enrichment, and *E. coli mcr*A^+^ cells, the MBs are liberated from the CPP (i.e., R9 in this study) and presumably hybridized to the specific target mRNA (i.e., *mcr*A in this study) inside the cells. In contrast, FISH-TAMB probes remain stable in cell-free conditions, wherein the CPPs remain non-covalently bound to MBs, keeping MBs from hybridizing to the specific target mRNA. While the exact mechanism remains unknown, the MBs released from R9s appear to retain hairpin conformation following cellular penetration, as evidenced by low fluorescence in the negative control *E*. coli *pmo*A^+^ cells incubated with FISH-TAMB probes (Fig. S4I). This procedural check provided the first remarkable sign that the *mcr*A FISH-TAMB probes labeled *mcr*A-expressing archaeal and bacterial populations.

### Step 5: Applications of FISH-TAMB Probes

Here, we describe three examples as a demonstration of FISH-TAMB labeling of cells that expressed the target *mcr*A mRNA and its combined use with flow cytometry and fluorescence microscopy.

#### (1) Enumeration of FISH-TAMB Labeled Cells by Flow Cytometry

This experiment was performed to illustrate that living cells are labeled by FISH-TAMB due to the expression of the target mRNA. *E. coli* cultures were used as the subject of study because of their easy manipulation and relatively fast doubling time. In principle, *E. coli* cells each carrying the transformed plasmid should express the gene insert upon induction of the *lac*Z operon where the insertion site is located. Thus, induced and uninduced *E. coli mcr*A^+^ cells were subjected to mRNA detection by *mcr*A FISH-TAMB probes appended with a Cy5 fluorophore. *E. coli mcr*A^+^ cell suspensions were split into two equal volumes when the OD_600_ reached > 0.6. One of the halves was induced with isopropyl β-D-1-thiogalactopyranoside (IPTG) at a final concentration of 1 mM. The cell suspensions, with or without IPTG addition, were incubated for 4 h more at 37 °C with shaking at 150 rpm. Afterward, optical density was measured again, and an appropriate volume (less than 1 μL) of uninduced and induced cells was taken for dilution to ~ 10^6^ cells/mL in 100 μL of 1 μM *mcr*A FISH-TAMB probe working solution (equivalent to 100 pmol MBs in 1 × DPBS). (It was found that 1 × DPBS gave a significantly lower noise (event counts) in flow cytometry analysis than home-made PBS solution.) The reactions without FISH-TAMB probes were used to set gates for cell populations.

In addition, non-specific target controls were included to reveal the number of cells labeled as a result of potentially non-specific hybridization: 1 μM *mcr*A FISH-TAMB probe were applied to IPTG-induced *E. coli lac*Zα^+^ cells (i.e., pGEM-T::*lac*Zα, without a *mcr*A gene insert), and 1 μM *lac*Zα FISH-TAMB probes were applied to IPTG-induced *E. coli mcr*A^+^ cells (i.e., without an intact *lac*Zα gene). IPTG induced cells were prepared as described above. For background subtraction, cell-free controls were set up to collect fluorescence signals from the unbound *mcr*A or *lac*Zα FISH-TAMB probes in the buffer solution, which contains 1 μM *mcr*A or lacZα FISH-TAMB probe in 1 × DPBS, plus 1 μL Luria Broth containing 0.05 mg/mL ampicillin (LB/A).

All reactions were prepared in triplicates. Following incubation at 37 °C for 15 min on a thermal cycler, reaction mixtures were diluted in 0.9 mL 1 × DPBS solution containing Fluoresbrite™ plain red 0.5 μm microspheres (Polysciences, Inc., Warrington, PA, USA) at the final concentration of ~ 10^5^ microspheres/mL, approximating to the cell concentrations. Fluorescent microsphere counts were used to calculate the volume of fluids analyzed, which was then used to determine the actual cell concentrations. All reactions were kept on ice and in the dark until analyzed to reduce cellular activity and photodegradation of fluorophores.

Flow cytometry was performed on a BD LSRII Multi-Laser Analyzer (BD Biosciences, San Jose, CA, USA) at the Princeton University Flow Cytometry Resource Facility. Data were acquired for 120 s for each sample at 8 μL/min average flow rate using four independent laser channels at default wattage settings (355 nm at 30 mW, 405 nm at 50 mW, 488 nm at 20 mW, and 640 nm at 40 mW). Forward and side-scattered light were set to logarithmic scale and used to trigger events. The system was flushed with 10% (v/v) bleach solution for 1 min followed by de-ionized water for 1 min before and after analysis and between samples to minimize the potential for cross-contamination.

Cell-sized objects (hereafter called “cells”) were gated with respect to the side-scattered light area and fluorescence signals of microspheres along the 575/26 nm (PE) bandpass filter. This gate was sufficient to identify *E. coli* cells based on known autofluorescence properties [35]. FISH-TAMB-labeled cells were identified as appropriately auto-fluorescent cells that also demonstrated at least a 10% increase in fluorescence on a 670/30 nm (Cy5) bandpass filter relative to the non-FISH-TAMB-treated cell populations. Gating was performed using BD FACSDiva v8.0.1 software (BD Biosciences, San Jose, CA, USA). A number of events gated as “cells” and “FISH-TAMB-labeled cells” in the cell-containing samples were determined, from which subtracted the number of corresponding events gated in the respective cell-free controls containing *mcr*A or *lac*Zα FISH-TAMB probes. Statistical analysis of observed differences in FISH-TAMB labeling between pairs of samples was performed using Student’s t-test in Microsoft Excel.

Flow cytometry results were supplemented by examination under confocal fluorescence microscopes, including an attempt for 3D imaging of FISH-TAMB labeled cells (Video S1). The description is provided in the next section and Supplementary Materials and Methods. Raw flow cytometry data and microscopy images are available upon request.

#### (2) Visualization of FISH-TAMB Labeled Cells by Fluorescence Microscopy

This experiment was performed to illustrate that cells at different transcriptional states could be revealed by FISH-TAMB labeling. Changes in the growth environment trigger microbial response within individual cells and across the cell population. Cells at exponential phase, and when stressed, are known to enter, broadly speaking, different metabolic states or, more specially, different transcriptional states. *M. barkeri* cells were used partly because they are a natural host of *mcr*A gene and partly because the strict anaerobe is easily stressed by exposure to O_2_ in the air.

*M. barkeri* cells were harvested at the exponential phase as determined by OD_550_ readings. An appropriate volume (50 μL) of *M. barkeri* cells was taken for dilution to ~ 10^6^ cells/mL in 100 μL of 1 μM *mcr*A FISH-TAMB (Cy5) probe working solution (equivalent to 100 pmol MBs) prepared using degassed 1 × DPBS in an anaerobic glove bag (Coy Laboratory Products, Grass Lake, MI, USA) to maintain cell activity in the absence of atmospheric O_2_ and incubated for 15 min at 37 °C. In addition, to assess the transcriptional state of *M. barkeri* under stress, *M. barkeri* cells were exposed to atmospheric O_2_ by transferring 1-mL aliquots (~ 10^8^ cells) into sterile 1.5-mL Eppendorf tubes and incubated under aerobic condition at 37 °C overnight with shaking at 150 rpm. Exposure to O_2_ was verified via media color change from clear (anaerobic) to bright pink (oxidized) as indicated by O_2_ sensitive resazurin in solution. FISH-TAMB hybridization was performed as described above.

FISH-TAMB-treated *M. barkeri* samples were imaged using an Olympus BX-60 microscope equipped with an MPlan 100 × magnification/0.90 BD Infinity objective, a 3 M-Pixel Digital Camera (Olympus), and Osram HBO Mercury burner (103 watts) and tungsten-halogen lamp (100 watts). The DAPI (352–477 nm) and Texas Red (633–738 nm) filters were applied when observing F420 autofluorescence of *M. barkeri* and the Cy5 fluorescence of FISH-TAMB probes, respectively. Composite micrographs were generated from raw microscopy images using ImageJ v. 2.0.0-rc-69/1.52n. Images were enhanced to show contrast using Adobe Photoshop Elements 15 (Adobe Inc., San Jose, CA USA).

Microscopic observations of *M. barkeri *cells from the exponential phase and that grown in contact with air were supplemented by flow cytometry. Following incubation with FISH-TAMB probes, reaction mixtures were diluted in 0.9 mL degassed 1 × DPBS solution containing Fluoresbrite™ plain red 0.5 μm microspheres (Polysciences, Inc., Warrington, PA, USA) at a final concentration of ~ 10^5^ microspheres/mL and then analyzed as described above.

#### (3) FISH-TAMB Labeling of Active ANMEs in an Enrichment Culture

This experiment was performed to illustrate that active, low-abundance populations are labeled by FISH-TAMB method. Active anaerobic methane oxidation (AOM) at the borehole BE326 BH2, a deep continental biosphere habitat, was due to the activity of uncultured ANMEs [8, 23]. We obtained an ANME consortia by supplement of ^13^CH_4_ and sulfate to BE326 BH2-Conc fracture fluid, and from the consortia, total DNA was extracted and analyzed for community composition. Metagenomic analysis was performed to confirm the presence of ANME-2 lineages and their relative abundance. The methodology for enrichment, DNA extraction, and metagenomic analysis are detailed in the Supplementary Materials and Methods.

FISH-TAMB hybridization followed by spinning-disk confocal microscopy and flow cytometry were performed. After 50 days of incubation, cell concentration was determined by OD_550_ measurements, and cells were harvested. An appropriate volume (50 μL) of cells was taken for dilution to ~ 10^6^ cells/mL in 100 μL of 1 μM *mcr*A FISH-TAMB (Cy5) probe working solution (equivalent to 100 pmol MBs) prepared using degassed 1 × DPBS in an anaerobic glove bag to maintain cell activity in the absence of atmospheric O_2_. After incubation at 37 °C for 15 min on a thermal cycler, reaction mixtures were transferred to individual wells of a Cellvis chambered cover glass and imaged using a Nikon Ti-E with an inverted microscope (Nikon Instruments, Melville, NY USA) equipped with the Perfect Focus System (PFS), a 100 Plan Apo (NA=1.45) oil objective lens, Yokogawa CSU-21 spinning disk, and Orca Flash camera (Hamamatsu, Bridgewater, NJ, USA). The 405-nm laser channel was used to excite F420 autofluorescence of ANME (excitation 405 nm, emission 461 nm). Excitation and emission wavelengths of the Cy5 fluorophore in FISH-TAMB probes were set to 647 nm and 670 nm, respectively. Instead of taking still images, live-cell time-lapse imaging data was acquired every min for 14.5 h, with multiple positions recorded simultaneously using an MS-2000 motorized stage (Applied Scientific Instrumentation, Eugene, OR, USA). It is specially noted that PFS on the Nikon Ti-E is a unique hardware solution designed to combat axial focus fluctuations in real time during long-term imaging investigations, which monitors and maintains the distance between the objective and the specimen, and has a response time in milliseconds.

To constrain the taxonomic identity of the FISH-TAMB-labeled cells in this ANME consortia, the 16S rRNA FISH probe EelMS-932 (5′-AGCTCCACCCGTTGTAGT-3′) targeting the ANME-2 subpopulation [36, 37] was used. A set of FISH-TAMB-treated cells were prepared from the ANME enrichment as mentioned earlier and were then fixed following an established FISH protocol [38]. Briefly, the FISH-TAMB-treated cells were centrifuged at 2,000 g for 5 min and washed once in 1 × DPBS. The supernatant was pipetted off, and cells were resuspended in a 1:1 mixture of chilled absolute ethanol and 1 × DPBS and stored overnight at -20 °C before subsequent filtration onto a 0.2-μm polycarbonate membrane filter (Whatman International Ltd., Maidstone, UK). Filters were washed twice with filter-sterilized distilled MilliQ water and then once with chilled absolute ethanol and then allowed to air-dry before being stored at -20 °C until 16S rRNA FISH treatment.

For traditional 16S rRNA FISH method, filter sections (containing fixed cells) were cut with flame-sterilized razor blades and placed on glass slides. Hybridization of fixed cells were performed using 50 ng/μL of dual-labeled EelMS-932, with an Atto 565 fluorophore at both the 5′- and 3′-end for stronger fluorescence signal (Biomers.net, Ulmer, Germany; ATTO-TEC GmbH, Siegen, Germany) in 2 mL hybridization buffer containing 900 mM NaCl, 20 mM Tris–HCl at pH 7.4, and 0.01% w/v SDS. Formamide was added to a final concentration of 40% (v/v) according to previously established hybridization stringency assessments [36]. The 16S rRNA FISH was performed by carefully covering slides with the hybridization mixture and sealing them inside 50-mL Falcon tubes (Corning Inc., Corning, NY USA) as “humidity chambers” containing a moist Kimwipe (Kimberly-Clark, Irving, TX USA) that had been soaked in the hybridization buffer. Hybridization proceeded at 46 °C for 2 h before slides were taken out of the humidity chambers and subsequently washed with washing buffer (60 mM NaCl, 20 mM Tris–HCl at pH 7.4, 5 mM EDTA, 0.01% w/v SDS). Slides were carefully covered with the washing buffer and incubated at 48 °C for 15 min inside a 50-mL Falcon tube containing a moist Kimwipe that had been soaked in the washing buffer. Upon removal, slides were washed twice with distilled MilliQ water and left to air dry at room temperature before being counterstained with 1 μM 4,6-diaminidino-2-phynylindole (DAPI) following an established protocol [38].

Flow cytometry was performed on a BD LSRII Multi-Laser Analyzer (BD Biosciences, San Jose, CA, USA) as described earlier. Data were acquired for 120 s for each sample at 8 μL/min average flow rate using four independent laser channels at default wattage settings (355 nm at 30 mW, 405 nm at 50 mW, 488 nm at 20 mW, and 640 nm at 40 mW). Forward and side-scattered light were set to logarithmic scale and used to trigger events. The system was flushed with 10% (v/v) bleach solution for 1 min followed by de-ionized water for 1 min before and after analysis and between samples to minimize the potential for cross-contamination. *M. barkeri* and methanogenic BE326 BH2-Conc cells were identified as sub-populations from the cell-sized objects gate on a 450/50 nm (F420) filter (321 V) that measures autofluorescence of the F420 enzyme (420-nm emission) [39]. FISH-TAMB-labeled cells were identified as appropriately auto-fluorescent cells that also demonstrated at least a 10% increase in fluorescence on a 670/30 nm (Cy5) bandpass filter relative to the non-FISH-TAMB-treated cell populations. Gating was performed using BD FACSDiva v8.0.1 software (BD Biosciences, San Jose, CA, USA). A number of events gated as “cells” and “FISH-TAMB-labeled cells” in the cell-containing samples were determined, from which subtracted the number of corresponding events gated in the respective cell-free controls containing *mcr*A FISH-TAMB probes. Statistical analysis of observed differences in FISH-TAMB labeling between pairs of samples was performed using Student’s t-test in Microsoft Excel.

### Growth Assessment of FISH-TAMB-Treated Cells

The fixation-free FISH-TAMB protocol raises the possibility of cultivating FISH-TAMB-treated cells in the laboratory. To assess the viability of the cells after FISH-TAMB treatment, growth curve analysis was performed on FISH-TAMB-treated cells.

An appropriate volume of *E. coli mcr*A^+^, *E. coli lac*Z${\alpha }$^+^, and *M. barkeri* were diluted to ~ 10^6^ cells/mL in 100 μL of 1 μM *mcr*A FISH-TAMB (Cy5) probe working solution (equivalent to 100 pmol MBs), and hybridization incubation was performed as described above for respective cultures. Subsequently, *E. coli* cells were inoculated into 2 mL of aerobic LB/A and *M. barkeri* into anaerobic DSMZ 120a media. Growth curves were obtained by monitoring OD_600_ for *E. coli*, and OD_550_ for *M. barkeri.* A set of reactions without FISH-TAMB probes and of blank media was done in parallel and served as the controls. Optical density was converted to cell concentration (per mL) using conversion factor, 8 × 10^8^ cells for one OD_600_ unit and 1.03 × 10^9^ cells for one OD_550_ unit. Growth rates (µ) were determined from the exponential phase of cellular growth plotted on logarithm scale and were compared between cultures treated with and without FISH-TAMB probes. Doubling time was calculated from the growth rates using this equation: LN(2)/µ.

## Results and Discussion

### FISH-TAMB Labels Intracellular mRNA Targets

*E. coli* strains have been used as the workhorse for protein (over)expression because transcription and subsequent translation expression are easily manipulated. Taking advantage of the established system for turning on and off the gene introduced through plasmid transformation, our FISH-TAMB probes can be tested under a controlled gene expression environment.

**Fig. 2:**
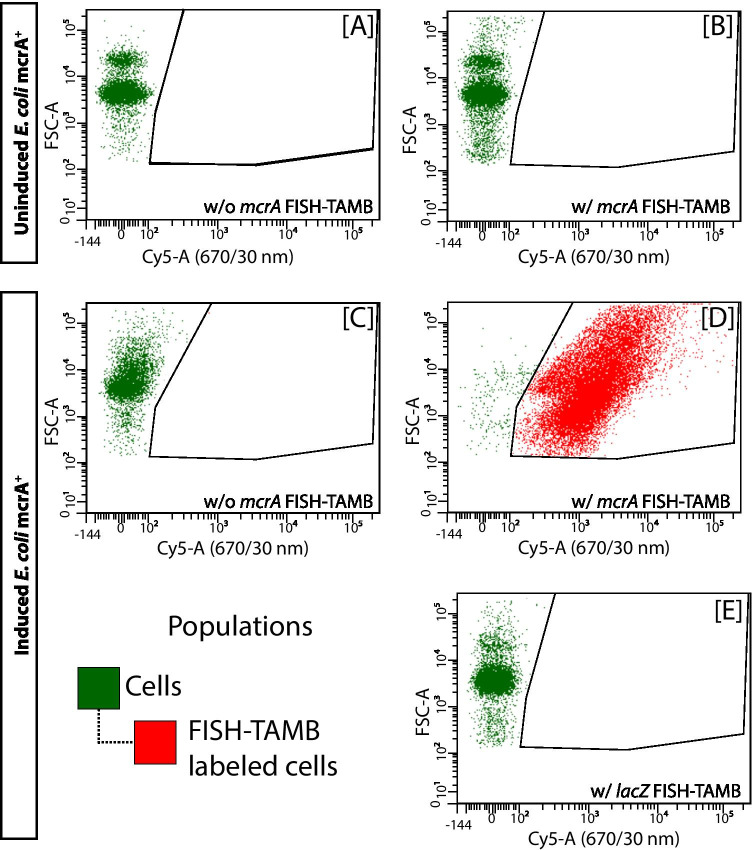
Flow cytometry data of FISH-TAMB targeting mRNA in *E. coli* grown in the absence (uninduced) or presence (induced) of IPTG, which triggers the transcription of the lac operon containing this gene. Events with optical properties similar to as *E. coli* cells are gated in green as cells. FISH-TAMB targeting *mcr*A mRNA in induced *E. coli* mcrA^+^ is indicated by the population gated in red. Cy5 was excited at 640 nm and emitted fluorescence collected via 670/30 nm bandpass filter. FSC-A stands for the area of forward-scattered density. **A** Uninduced *E. coli mcr*A^+^ without FISH-TAMB treatment. **B** Uninduced *E. coli* mcrA^+^ treated with FISH-TAMB probes targeting *mcr*A mRNA. **C** IPTG-induced *E. coli* mcrA^+^ without FISH-TAMB treatment. **D** IPTG-induced *E. coli* mcrA^+^ treated with FISH-TAMB probes targeting *mcr*A mRNA. **E** IPTG-induced *E. coli* mcrA^+^ treated with FISH-TAMB probes targeting *lac*Z ${\alpha }$ mRNA

In the absence of the expression-inducing agent IPTG, flow cytometry showed that a negligible proportion (0.00 ± 0.02%) of uninduced *E. coli mcr*A^+^ cells were assigned as FISH-TAMB-labeled cells (﻿Fig. 2B; Table 1), which was not statistically different from the FISH-TAMB-free controls containing uninduced *E. coli mcr*A^+^ (-0.01 ± 0.03%) (Fig. 2A; Table 1) (Student’s t-test, t = -0.12, *p* = 0.91). Barely negative values in Table 1 are due to background subtraction of corresponding events in cell-free blanks, among which one replicate had “FISH-TAMB-labeled cells” detected. Nonetheless, these false positives were within the error of measurement. These two treatments should both be understood as containing 0% of FISH-TAMB-labeled cells, indicating that the basal expression of the target *mcr*A gene was generally too low for individual cell detection by FISH-TAMB. By contrast,﻿ the vast majority (92.48 ± 5.65%) of the induced *E. coli mcr*A^+^ cells were labeled by FISH-TAMB (Fig. 2D; Table 1), when compared to 0.03 ± 0.01% in the parallel samples without FISH-TAMB treatment (Fig. 2C; Table 1), indicating that cells expressing *mcr*A upon IPTG induction were detectable by the FISH-TAMB method. These results also indicated that the detected Cy5 signals in the induced *E. coli mcr*A^+^ samples were not attributed to IPTG autofluorescence. In addition, even though *E. coli* JM109 and the pGEM-T Easy Vector are a cloning system for DNA propagation, upon induction, the high-copy number of plasmids per cell yielded a sufficiently high *mcr*A mRNA expression level for FISH-TAMB detection.

**Table 1:** Flow cytometry data of cell cultures

Cells	FISH-TAMB probes	Cell density (× 10^6^ per mL) [mean]	Cell density (× 10^6^ per mL) [SD]	No. of FISH-TAMB labeled cells# (10^6^ per mL) [mean]	No. of FISH-TAMB labeled cells# (10^6^ per mL) [SD]	% of FISH-TAMB labeled cells# [mean]	% of FISH-TAMB labeled cells# [SD]	MB probe (pmol per cell)^ [mean]	MB probe (pmol per cell)^ [SD]
Uninduced *E.coli mcr*A + (*n* = 3)	*mcr*A	0.340	0.036	0.000	0.000	-0.01%	0.03%	2.96E-04	3.01E-05
Uninduced *E.coli mcr*A + (*n* = 3)		0.277	0.044	0.000	0.000	0.00%	0.02%	3.67E-04	5.41E-05
Induced *E.coli mcr*A + (*n* = 3)	*mcr*A	3.805	2.274	3.602	2.363	92.48%	5.65%	3.40E-05	2.07E-05
Induced *E.coli mcr*A + (*n* = 3)		0.320	0.060	0.000	0.000	0.03%	0.01%	3.20E-04	6.06E-05
Induced *E.coli mcr*A + (*n* = 3)	*lac*Za	0.325	0.083	0.000	0.000	0.01%	0.02%	3.20E-04	7.82E-05
Uninduced *E.coli lac*Za + (*n* = 3)	*lac*Za	0.276	0.112	0.155	0.082	54.13%	7.19%	4.09E-04	1.75E-04
Induced *E.coli lac*Za + (*n* = 3)	*lac*Za	0.879	0.386	0.638	0.253	74.15%	7.04%	1.33E-04	6.77E-05
*M. barkeri* at exponential phase (*n* = 3)	*mcr*A	0.023	0.010	0.006	0.002	28.83%	4.76%	4.70E-03	1.64E-03
*M. barkeri* exposed to O2 (*n* = 3)	*mcr*A	0.001	0.001	0.000	0.000	1.76%	3.62%	7.82E-02	3.67E-02
ANME enrichment (*n* = 3)	*mcr*A	0.049	0.015	0.001	0.000	2.62%	0.19%	2.05E-03	5.47E-04

^#^Background subtracted

^^^The amount of MB probes was calculated by dividing the cell density (counted by flow cytometry) by the amount of MB (in the form of FISH-TAMB) probes added to the reaction mixture

The result of uninduced and induced *E. coli lac*Za^+^ cells was described and discussed in the Supplementary Results and Discussion

When the induced *E. coli mcr*A^+^ cells were treated with *lac*Zα FISH-TAMB probes that target the insertion site, only a very small percentage (0.01% ± 0.02%) of cells was assigned as FISH-TAMB-labeled (Fig. 2E; Table 1). This result indicated that the majority of the *mcr*A FISH-TAMB-labeled cells did carry an insert that has disrupted the insertion site and it was the *mcr*A gene insert that resulted in Cy5 fluorescence.

Thanks to flow cytometry that provides a detailed account for gene expression at the single-cell level, these results indicated that FISH-TAMB, when applied at 10^–3^ to 10^–5^ pmol probes per cell (Table 1), detects with high confidence cells that expressed the target mRNA at a considerable level. Further investigation is required to express quantitatively the sensitivity of FISH-TAMB and flow cytometry, such as the minimal copy numbers of the target mRNA per cell for discernable signal-to-background fluorescence.

### FISH-TAMB Differentiates Transcriptional Levels

**Fig. 3:**
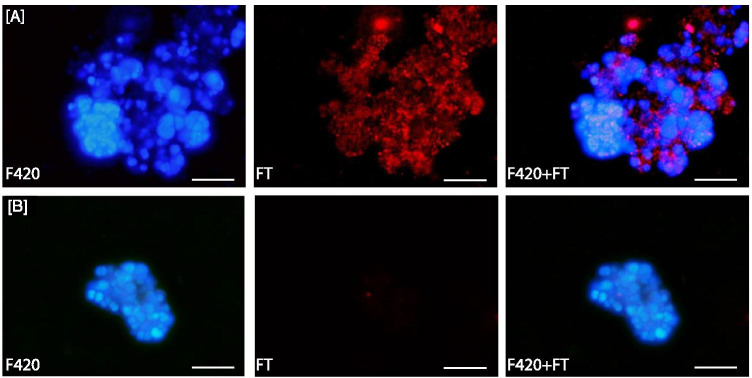
FISH-TAMB sensitivity to *mcr*A transcription in *Methanosarcina barkeri* during **A** exponential phase, and **B** following overnight exposure to air*.* F420, F420 autofluorescence; FT, Cy5 fluorescence from FISH-TAMB labeling; F420 + FT, composite image of F420 and FT micrographs. Scale bar 10 µm

*M. barkeri* conserves energy through hydrogenotrophic methanogenesis when grown using H_2_ as the sole electron donor. As *M. barkeri* cells enter different growth stages due to substrate limitation in batch culture, or experience stressful conditions such as exposure to O_2_, their investment on the energy conservation machinery is anticipated to adjust accordingly, resulting in variation in the expression of *mcr*A gene. Coupling FISH-TAMB with epifluorescence microscopy, we observed remarkable variability in Cy5 fluorescence level between *M. barkeri* cells during exponential phase and after O_2_ exposure (Fig. 3).

During exponential growth phase, the formation of aggregates resulted in only ~ 28% of FISH-TAMB-labeled *M. barkeri* cells (Table 1), even though all observable cells from the exponential phase detected from F420 autofluorescence were co-labeled with FISH-TAMB probes targeting *mcr*A mRNA (Fig. 3A). Three main reasons have contributed to the apparent absence of autofluorescence in some FISH-TAMB-associated fluorescence areas: (1) *M. barkeri* cell’s autofluorescence is due to oxidized coenzyme F420, and *M. barkeri* cells are known to form aggregates. It is believed that the inner cells were less prone to ambient O_2_; in other words, it would take longer for the coenzyme F420 in the inner cells to be oxidized; (2) brightness/contrast of the images from blue and red channels was adjusted separately, resulting in a more prominent red coverage than blue; and (3) blue and red channel micrographs were captured at slightly different focal planes so that each channel’s image showed the most detail in focus.

By comparison, following overnight exposure to atmospheric O_2_, FISH-TAMB-associated fluorescence was absent (Fig. S5) or appeared only near the center of the aggregate (Fig. 3B) and accounted for fewer than 2% of all observed cells. Both the decrease in the number of labeled cells and observed drops in Cy5 fluorescence are consistent with anticipated diminished methanogenesis rates typical of prolonged O_2_ exposure [40]. The spatial pattern of the *mcr*A-transcribing vs. *mcr*A-non-transcribing cells also reflected the spatial zonation or heterogeneity of cell aggregates in relationship to nutrient availability and presence of inhibitory substances [41]. The results suggested that a number of cells labeled by FISH-TAMB appear to be affected by the mRNA expression level, although further experiments will be necessary to test this hypothesis and quantitatively evaluate the FISH-TAMB fluorescence and mRNA copy number.

### FISH-TAMB Detects Active, Rare Taxa

Time-lapse fluorescence imaging has revolutionized our understanding of cellular activity and compartmentalization [42, 43]. However, real-time tracking of RNA and protein tagged by fluorescent molecules is done, to our best knowledge, on model or engineerable organisms in axenic (co-)cultures. If a similar methodological approach is available for studying uncultured microorganisms, regardless of their abundance (of course, it will be more challenging for the rare taxa), it is foreseen that cellular responses and perhaps physiological and behavioral aspects of uncultured microorganisms can be unveiled. A non-lethal and non-destructive labeling or tagging method would be one of the keys for realizing real-time live-cell imaging of uncultured microorganisms, ideally in environmental samples in the laboratory or even in situ in the field.

AOM activity of the enrichment culture from the borehole BE326 BH2 was confirmed by observation of ^13^CH_4_ conversion to ^13^CO_2_. About 3% of cells were labeled by the *mcr*A FISH-TAMB probes, which potentially had hybridized to *mcr*A mRNA in methanogens and ANMEs (Table 1; Fig. S6). Metagenomic analysis confirmed the presence of two putatively novel ANME-2d lineages within the archaeal family *Methanoperedenaceae*, collectively comprising 1% relative abundance among the *Archaea* domain and 0.03% abundance relative to the entire microbial community. Recent evidence suggested that ANME-2d are capable of performing AOM coupled to sulfate reduction [44], though it is not clear whether they are capable of carrying out this process alone or a syntrophic partner is required for electron exchange. Sequences assigned to canonical ANME sulfate-reducing bacterial partners—*Desulfosarcina/Desulfococcus* (0.08%), *Desulfobulbus* (0.05%), and *Desulfotomaculum* (1%) [45, 46] were also detected, though at this time it is unclear if any of these groups partners metabolically with the identified ANME-2d lineages.

**Fig. 4:**
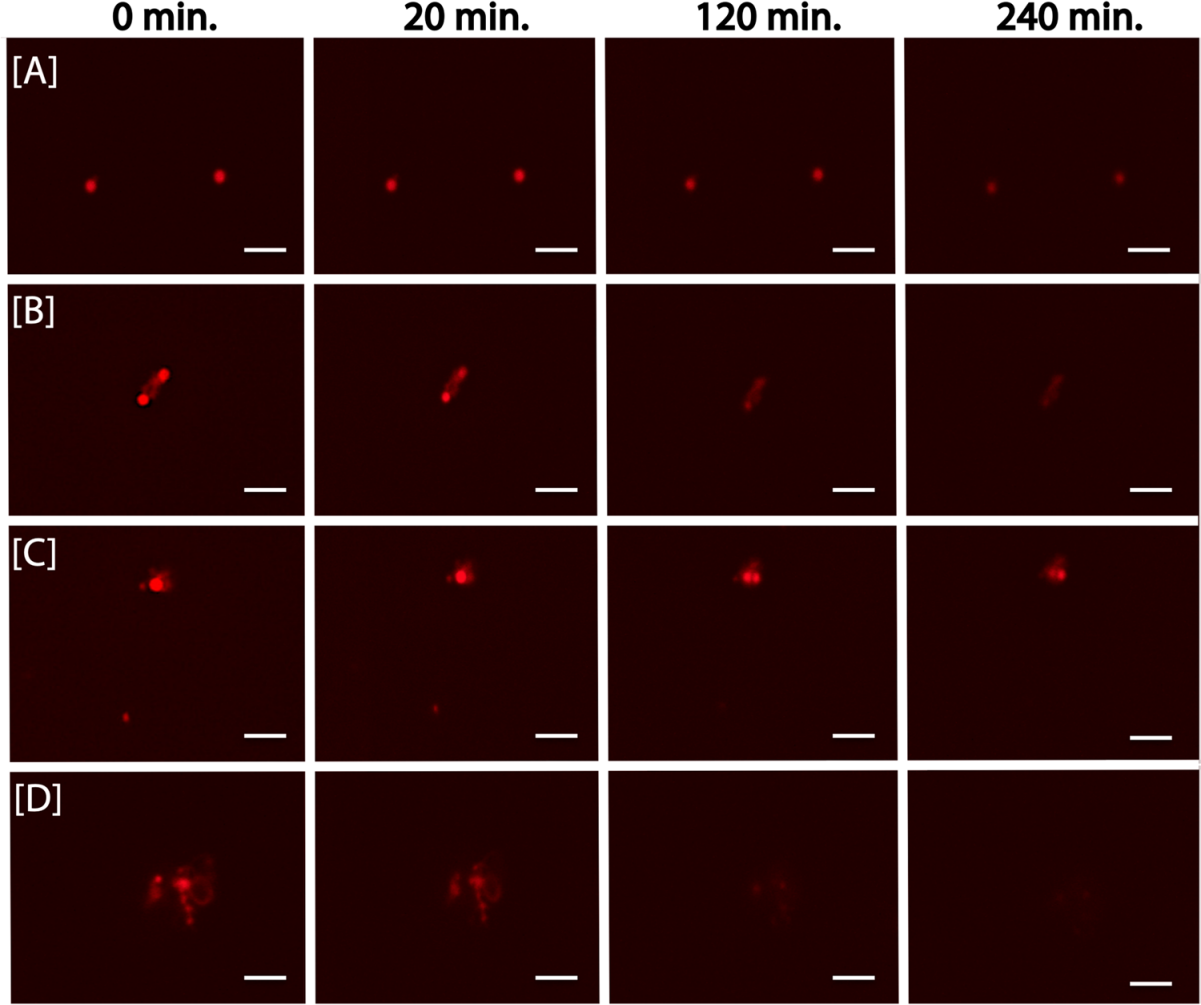
Snapshots of time-lapse microscopy of ANME enrichment culture labeled by mcrA FISH-TAMB probes. BE326 BH2 ANME enrichments were incubated anaerobically with 1 µM FISH-TAMB probes targeting *mcr*A mRNA and subsequently imaged via spinning-disk fluorescence confocal microscopy. Micrographs were snapped every minute for 14 h with an exposure of 100 ms. Composite micrographs of brightfield and Cy5 channel represent the first 4-h observation of **A** single cells; **B** and **C** physically associated cells; and **D** cell aggregate. Scale bar 5 µm

Using spinning-disk fluorescence confocal microscopy, we demonstrated the potential of using FISH-TAMB for live-cell imaging for a period of time. FISH-TAMB-labeled cells from the ANME enrichment were observed over the first 4 h of monitoring, and snapshots at 0, 20, 120, and 240 min were taken for single planktonic cells (Fig. 4A), paired cells (Fig. 4B and C), and cell aggregates (Fig. 4D). Interestingly, a cell duplet appeared to have undergone some changes over the course of observation, wherein a second labeled cell seemed to emerge from the original one of larger size (Fig. 4B and Video S2). Because of the short response time of the PFS of the microscope, it is unlikely that focus drift artificially generated the fluorescence signal by bringing an out-of-focus cell into view. The emergence of second labeled cell could therefore be explained by several scenarios: (1) rotation of two labeled cells into the focal plane, bringing an out-of-sight cell into view; (2) ongoing cell division during imaging, during which time the cells rotated into the focal plane, revealing a daughter cell with labeled *mcr*A mRNA adopted in the cytoplasm. Doubling times of sulfate-dependent AOM have been observed between 1.1 and 7.5 months [47], and as our ANME enrichment was incubated for 50 days, it would not be unexpected to observe dividing cells; or (3) the Cy5 fluorescence level in the second cell was initially below detection, and later it attained a detectable range. This curious finding warrants further experimentation to visually document the FISH-TAMB hybridization process to see how FISH-TAMB may be used for real-time labeling of newly formed target mRNA in the cytoplasm.

**Fig. 5:**
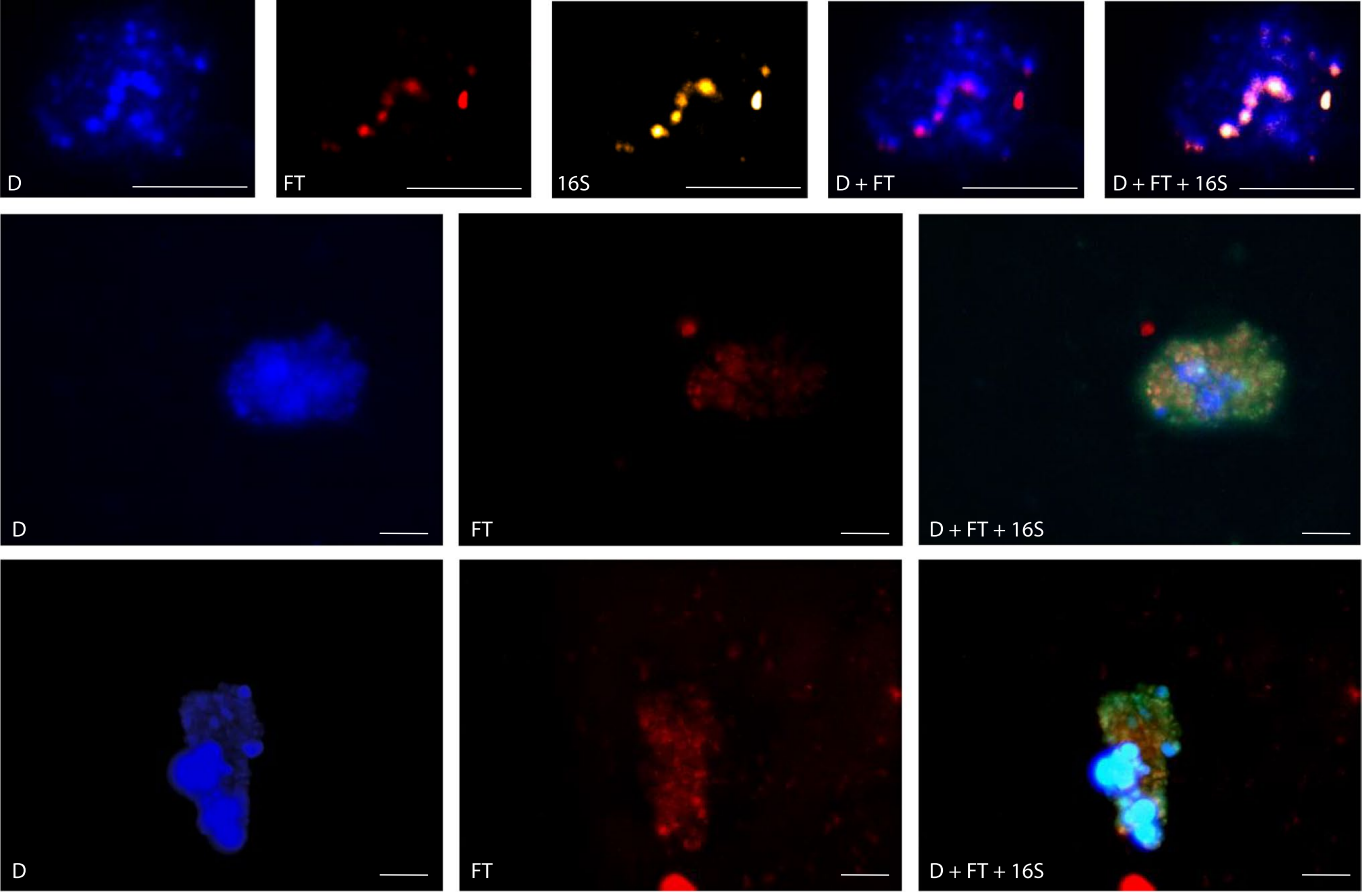
Co-labeling of active ANME-2 cells in ANME enrichment cultures by FISH-TAMB and 16S rRNA FISH. D, DAPI; FT, Cy5 fluorescence from FISH-TAMB labeling, 16S, Atto 565 fluorescence from 16S rRNA FISH labeling; D + FT + 16S, composite of D, FT, and 16S micrographs. Scale bar 10 µm

To establish the efficacy of FISH-TAMB as a method for tracking transcriptional activity of uncultivated lineages, AOM enrichment samples freshly treated with *mcr*A FISH-TAMB probes were subjected to the traditional (fixation required) 16S rRNA FISH protocol to identify ANME-2 archaea. Cells labeled by the *mcr*A FISH-TAMB probes were also labeled by the EelMS-932 probes, confirming that the *mcr*A FISH-TAMB-labeled cells include ANME-2, an active minority group. The ANME-2 cells were situated in consortia with DAPI-only labeled cells (Fig. 5), which were potentially the syntrophic partner sulfate-reducing bacteria (SRB) [8, 23] identified in the metagenome. These results provided the first microscopic evidence of active AOM-performing microbial consortia from the continental deep biosphere.

### FISH-TAMB Shows Little Impact to Cell Viability

**Fig. 6:**
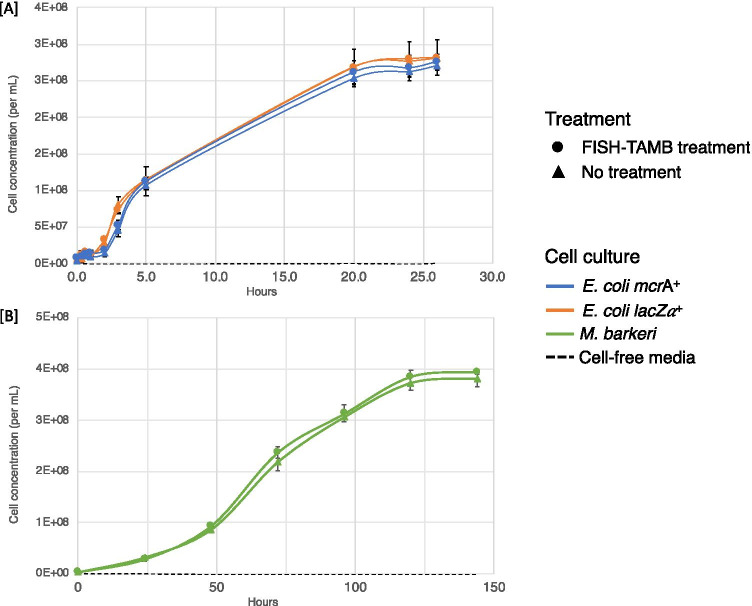
FISH-TAMB viability assessment by growth curve analysis. Pure cultures of **A** *E. coli mcr*A^+^ and *E. coli lac*Zα^+^ and **B** *M. barkeri* (~ 10^6^ cells mL^−1^) were incubated with 1 µM FISH-TAMB probes and inoculated into their respective growth media

To illustrate if FISH-TAMB treated cells remained culturable, we monitored the growth of *E. coli mcr*A^+^, *E. coli lac*Z $\alpha$^+^, and *M. barkeri* incubated with and without FISH-TAMB probes. All cultures treated with FISH-TAMB probes showed a similar duration of lag and exponential phases and entered the stationary phase at a similar time (Fig. 6). Both FISH-TAMB-treated and untreated *E. coli* exhibited similar growth rates (Fig. 6A). The specific growth rates of *E. coli mcr*A^+^ treated with or without *mcr*A FISH-TAMB probes were not significantly different from each other (Student’s t-test, t = 0.495, *p* = 0.65), with µ_FISH-TAMB_ = 1.07 ± 0.17 h^−1^ and µ_control_ = 1.14 ± 0.13 h^−1^, respectively. The specific growth rates of *E. coli lac*Z $\alpha$^+^ treated with or without *lac*Z $\alpha$ FISH-TAMB probes were also not significantly different from each other (Student’s t-test, t = 0.153, *p* = 0.89), with µ_FISH-TAMB_ = 0.99 ± 0.19 h^−1^ and µ_control_ = 1.01 ± 0.08 h^−1^, respectively. The corresponding doubling times ranged from 0.55 to 0.91 h. The specific growth rates for both control (µ_control_) and FISH-TAMB-treated (µ_FISH-TAMB_) *M. barkeri* were 0.04 ± 0.00 h^−1^ (Fig. 6B), which are consistent with previously reported values for hydrogenotrophic *M. barkeri* growth [48]. The corresponding doubling times ranged from 14.31 to 17.70 h. These results showed that the applied FISH-TAMB dosage (10^–3^ to 10^–5^ pmol MBs per cell as in Table 1) had no observable inhibitory effect on the growth of these pure cultures.

### Implications of FISH-TAMB for Microbial Ecology

FISH-TAMB utilizes polyarginine CPP to deliver MBs across prokaryotic cell walls and membranes, fluorescently labeling cells when MBs hybridize to target mRNA sequences. Hybridization occurs in isotonic buffer containing no fixatives or strong surfactants, at the (optimal) growth temperature of the tested cultures and enrichments, and in as short as 15 min. We demonstrated that FISH-TAMB labels intracellular mRNA targets, differentiates transcriptional states, detects active and rare taxa, and keeps cell viability. Coupling FISH-TAMB with various fluorescence detection approaches would enable qualitative and/or (semi-) quantitative studies of the target mRNA and its host cells, as well as physically associated cells exhibiting parasitic or syntrophic relationship. Although FISH-TAMB was initially envisioned for mRNA of any functional gene of interest without a requirement of prior knowledge of 16S rRNA-based taxonomy, FISH-TAMB has the potential for going beyond mRNA and multiplexing. Thus, FISH-TAMB is a versatile addition to the molecular ecologist’s toolkit, with potential widespread application in the field of environmental microbiology.

While FISH-TAMB provides the first step towards identifying rare, uncultured taxa as active players or keystone species in the deep biosphere and other ecosystems, continued development of FISH-TAMB is necessary to understand the limits of its application in other systems (e.g., temperature, salinity, and pH extremes; sensitivity to spore forming, gram-positive bacteria; double-membraned archaea [49]; the detection limit for transcript copy numbers; optimal, toxic, and lethal dosages respective to microbial lineages). Then, FISH-TAMB can truly enable us to characterize the genomes and physiologies of the labeled cells and subsequently to evaluate or perhaps even quantify their ecological importance.

## Supplementary Information

Below is the link to the electronic supplementary material.

Supplementary file1 (DOCX 1.70 MB)

Supplementary file2 (AVI 448 KB)

Supplementary file3 (AVI 486201 KB)

Supplementary file4 (MP4 211 KB)

## Data Availability

The sequence data of the BE326 BH2-Conc ANME enrichment metagenome generated and analyzed during the current study are available in the NCBI GenBank under accession number PRJNA562560 (Sequence Read Archive accession number SRR10029121).
