# Supplementary material for: FISH-TAMB, a Fixation-Free mRNA Fluorescent Labeling Technique to Target Transcriptionally Active Members in Microbial Communities
Source: Microb Ecol. 2021 Aug 18;84(1):182–97. doi: 10.1007/s00248-021-01809-5 (PMC9250922; doi:10.1007/s00248-021-01809-5)
Supplement: Supplementary file 1 — Supplementary file1 (DOCX 1.70 MB) [file 248_2021_1809_MOESM1_ESM.docx]

**SUPPLEMENTARY INFORMATION**

**FISH-TAMB, a fixation-free mRNA fluorescent labeling technique to target transcriptionally active members in microbial communities**

**Authors**: Rachel L. Harris, Maggie C. Y. Lau Vetter, Esta van Heerden, Errol Cason, Jan-G Vermeulen, Anjali Taneja, Thomas L. Kieft, Christina J. DeCoste, Gary S. Laevsky, and Tullis C. Onstott

This document contains

SI Materials and Methods

SI Results and Discussion

Figs. S1 – S6

Movies S1 – S2

Tables S1 – S2

SI References

**SI MATERIALS & METHODS**

***Methanosarcina barkeri* culture**

Freeze-dried *M. barkeri* cells (ATCC^®^ 43569^™^) were revitalized by inoculation into 9 ml of DSMZ medium 120a (pH 7.2). DSMZ medium 120a was prepared in an anaerobic glove bag (Coy Laboratory Products, Grass Lake, MI, USA) containing a 95:5 N_2_:H_2_ atmosphere. The following were added to one liter of degassed distilled H_2_O: 2 mM K_2_HPO_4_, 1.7 mM KH_2_PO_4_, 9.3 mM NH_4_Cl, 2 mM MgSO_4_ •7H_2_O, 1.7 mM CaCl_2_•2H_2_O, 2.25 g 4.3 mM NaCl, 2.00 ml of FeSO_4_•7H_2_O (0.1% w/v in 0.1 N H_2_SO_4_), 2.00 g yeast extract, 2.00 g casitone, 2.5 mM NaHCO_3_, 1.3 mM Na_2_S•9H_2_O, 1.7 mM L-cys HCl•H_2_O, 1.0 ml of trace element solution SL-10 (per liter of distilled H_2_O: 10.0 ml HCl [25%, 7.7 M], 1.50 g FeCl_2_•4H_2_O, 70 mg ZnCl_2_, 100 mg MnCL_2_•4H_2_O, 6 mg H_3_BO_3_, 190 mg CoCl_2_•6H_2_O, 2 mg CuCl_2_•2H_2_O, 24 mg NiCl_2_•6H_2_O, 36 mg Na_2_MoO_4_•6H_2_O), and 0.3 ml Na-resazurin solution (0.1% w/v). The medium was adjusted anaerobically to pH 7.2 with O_2_-free 1 M NaOH. The DSMZ medium 120a was then dispersed as 9mL-aliquots into 25 mL Balch tubes and sealed with butyl rubber stoppers (boiled in 0.1 N NaOH for 1 h; autoclaved) and aluminum crimps. The liquid medium was sparged for 20 minutes at 30 psi with 100% ultra-high purity Ar gas (Airgas, Inc., Radnor, PA USA) and subsequently autoclaved. MD-VS^™^ vitamin solution (ATCC^®^, Manassas, VA USA), 100 μL of 1% (v/v), was added to each vial after autoclaving, and the headspace was replaced and over-pressurized to 1.5X atmospheric pressure (atm) with 80:20 H_2_:CO_2_ gas (Airgas, Inc., Radnor, PA USA) by flushing for 15 min at 30 psi.

Cultures were transferred and maintained anaerobically in 160 mL borosilicate serum vials at 37˚C. H_2_ was the sole added electron donor. Growth at 37˚C was monitored via optical density measurements taken at 550 nm [OD_550_] [1] using a Hach DR/2010 Spectrophotometer (Hach Company, Loveland, CO, USA). Methane (CH_4_) production was observed using a gas chromatograph equipped with a flame ionization detector (FID) (Peak Performer 1 series, Peak Laboratories, Mountain View, CA, USA).

***E. coli* *mcr*A^+^ and *lac*Zα^+^ cultures**

The partial *mcr*A gene was originated from the *M. barkeri* culture. Triplicate 1-mL aliquots of *M. barkeri* (~10^9^ cells/mL) were centrifuged at 11,000 x g for 2 min. The supernatant was removed down to 10 μL and the cell pellet was stored at -80˚C prior to PCR amplification. Triplicate PCR amplifications were performed in 50 μL-reactions containing the thawed pellet, 1X PCR buffer, 200 μM dNTPs, 1% Tween-20, 1.5 U Taq DNA Polymerase (Takara Bio USA, Mountain View, CA, USA), 0.2 μM Mlas forward primer (5’-GGTGGTGTMGGDTTCACMCARTA-3’) modified from [2, 3], and 0.2 μM *mcr*A-rev reverse primer (5’-CGTTCATBGCGTAGTTVGGRTAGT-3’) modified from [3]. The polymerase chain reaction (PCR) was performed on a C1000 Touch^™^ Thermal Cycler (Bio-Rad Laboratories, Inc., Hercules, CA, USA) and consisted of a 5 min initial denaturation step at 94˚C and 35 cycles of the following: 1 min denaturation at 94˚C, 1 min annealing at 55˚C, and 1 min extension at 72˚C. Final extension lasted for 10 minutes at 72˚C. Positive amplification of ~500 bp amplicons was confirmed by gel electrophoresis and PCR products were purified by ethanol precipitation. Two volumes of pre-chilled absolute ethanol were added to each PCR reaction tube and mixed by inversion. Reaction tubes were incubated at -20˚C for 30 min, and subsequently centrifuged at 11,000 x g for 30 min. The supernatant was discarded, and the pellet was washed with 500 μl 75% ethanol prior to centrifugation at 11,000 x g for 5 min. The ethanol wash step was repeated once, the supernatant was discarded, and the pellet was resuspended in 1X TE buffer.

The purified *mcr*A PCR products were used to construct *E. coli* *mcr*A^+^ clones. Purified PCR products with 3’-A overhangs generated by Takara *Taq* DNA polymerase were ligated to pGEM^®^-T Easy vectors by incubating overnight at 4˚C and subsequently transformed into JM109 High Efficiency Competent *E. coli* cells by heat shock at 42 ˚C for 45 sec, according to the manufacturer’s instructions (Promega Corporation, Madison, WI, USA). Following transformation, *E. coli* were incubated for 1.5 hours at 37˚C in an orbital shaker at 150 rpm in a suspension of Luria Broth (LB) containing 0.05 mg/ml ampicillin (LB/A). *E. coli* cell density was measured via optical density at 600 nm [OD_600_] using a Beckman DU^®^ 530 Life Science UV/Vis Spectrophotometer (Beckman Coulter^®^, Indianapolis, IN, USA). The transformed cells of ~3.4 x 10^8^ cells/ml were serially diluted to ~10^2^ cells/mL and plated on an LB agar plate with 0.05 mg/mL ampicillin, 0.05 mg/mL IPTG, and 0.08 mg/mL X-gal for blue/white screening, and incubated overnight at 37˚C. White colonies were picked from the plate and inoculated into LB/A medium. Sanger sequencing of the plasmid confirmed orientation of the *mcr*A insert. *E. coli* clones carrying a correctly inserted *mcr*A gene, with its mRNA expression regulated by the T7 RNA polymerase promoter, grown from single colonies were labeled as *E. coli* *mcr*A^+^ cells. *E. coli mcr*A^+^ was periodically monitored for gene loss by plating onto LB agar plates. If no white colonies appeared, the cloning procedure was repeated.

Blue colonies contained a plasmid with an intact *lac*Zα gene, thus an undisrupted beta-galactosidase-encoding insertion site available for the hybridization of the *lac*Zα MB. Single colonies also picked from the plate and inoculated into LB/A medium. They are labeled as *E. coli* *lac*Zα^+^ cells.

***E. coli pmo*A^+^ expression clone transformation**

Total DNA extracted from BE326 BH2 fracture fluid collected in 2011 using 2x CTAB lysis buffer and phenol/chloroform [4] was used as template for amplification of partial *pmo*A gene . PCR amplification was performed as described above for *mcr*A gene amplification, except that 0.4 μM forward primer A189m (5’-GGNGAYTGGGACTTYTGG-3’) and 0.4 μM reverse primer A682m_a (5’-GAAYSCNGARAAGAACGM-3’), modified from Holmes *et al*. and Luesken *et al*. respectively [5, 6] were used. Positive PCR amplification was confirmed by gel electrophoresis, PCR products were purified and ligated to pGEM^®^-T Easy Vectors and transformed into JM109 competent cells, *E. coli* *pmo*A^+^ were isolated as white colonies, and maintained according to the procedure described above for other *E. coli* cultures.

**Anaerobic Methanotrophs (ANMEs) Enrichments**

Fracture fluid was collected in June 2016 following established sampling procedures [4, 7] from a horizontal borehole located 1.34 km below land surface on the 26^th^ level of shaft 3 of the Beatrix Gold Mine in South Africa (BE326 BH2) (S 28.235º, E 26.795º). Due to low *in situ* cell concentration of 10^3^ to 10^4^ cells/mL [8] the fracture fluid was first filtered using a 0.2 μm hollow fiber MediaKap^®^-10 filter (Spectrum Labs, New Brunswick, NJ USA) and then back-flushed with fracture fluid into sterile, N_2_-sparged 160 mL borosilicate serum vials to obtain a final concentration of ~10^7^ cells/mL (later referred to as BE326 BH2-Conc) . Dissolved gas samples were collected along with field measurements of certain environmental parameters. The DSMZ medium 120a was then dispersed as 9 mL-aliquots into 25 mL Balch tubes and sealed with butyl rubber stoppers (boiled in 0.1 N NaOH for 1 h; autoclaved) and aluminum crimps.

For enrichment of sulfate-dependent anaerobic methanotrophy (S-AOM), 10 mL of BE326 BH2-Conc fracture fluid was added to 90 mL modified artificial seawater medium in 160 mL borosilicate serum vials [9, 10]. Per 1 liter of distilled, degassed H_2_O was 376 mM NaCl, 49 mM MgCl_2_, 10 mM Na_2_SO_4_, 8.5 mM KCl, 2.5 mM NaHCO_3_, 1.3 mM Na_2_S•9H_2_O, 1.7 mM L-cys HCl•H_2_O, 1 ml of SL-10 trace metal solution (as described above in *M. barkeri* cultivation), 10 ml of MD-VS^™^ vitamin solution, 0.3 ml (0.1% w/v) Na-resazurin solution, final pH 8.2. The medium pH and 10 mM SO_4_^2-^ was referenced to the *in-situ* conditions observed at BE326 BH2 (Table S1). Media sterilization, addition of vitamins, and anaerobic sparging followed the protocol described above for DSMZ 120a medium, with the exception that serum vial headspaces comprised 2% ^13^CH_4_ (Isotec® Stable Isotopes, Millipore Sigma, St. Louis, MO USA) in a balance of ultra-high purity N_2_ gas (Airgas, Inc., Radnor, PA USA).

AOM activity was determined via stable isotopic monitoring of ^13^CH_4_ tracer oxidized to ^13^CO_2_ using a Picarro G2101-*I* cavity ringdown spectrometer (Picarro, Inc. Santa Clara, CA USA). Sulfate reduction to sulfide was monitored using a Dionex IC25 ion chromatograph coupled to an MSQ-quadruple mass spectrometer (Thermo Scientific, Waltham, MA USA).

Total DNA isolation was performed on the BE326 BH2-Conc S-AOM enrichment for metagenomic sequencing using a Qiagen DNeasy PowerSoil Kit following the manufacturer’s protocol (QIAGEN, Hilden, Germany). Metagenomic libraries were prepared using a PrepX DNA library kit and an automated Apollo 324 system (WaferGen Biosystems, Inc., Fremont CA USA). Paired-end (2× 100 nt) DNA sequencing was performed on a HiSeq 2000 platform (Illumina, Inc., San Diego, CA USA) located at the Marine Biological Laboratory in Woods Hole, MA USA. Quality filtering of sequenced reads and subsequent metagenome assembly and annotation was performed as previously described [11]. A total of 35,669,635 raw paired-end reads were processed using fastp v.0.12.6 [12] to remove reads matching the Illumina universal adapter sequence, that were shorter than 50 nt, had Phred quality scores < 30, and contained Ns as bases. The resulting 32,139,898 quality-filtered paired-end reads were assembled using SPAdes v.3.11.0 (-meta option) [13]. Taxonomic diversity of the assembled metagenome was assessed using Kaiju v.1.6.2 [14] against the nr_euk database (including fungi and microbial eukaryotes) using greedy mode (-a greedy), allowing 5 mismatches (-e 5) and a maximum e-value threshold of 5×10^-5^ (-E 0.00005).

**Swept-field confocal microscopy for 3D imaging**

To explore the possibility of imaging the localization of mRNA in living cells, a demo section was offered by Bruker Scientific Instruments. Cells treated with *mcr*A and *lac*Zα FISH-TAMB probes, respectively, were live-stained with 1 µM Hoechst 33342 and imaged using a Bruker Opterra II swept-field confocal microscope (Bruker Scientific Instruments, Billerica, MA, USA). Hoechst 33342 fluorescence was excited with a 405 nm laser line and emission was detected at 461 nm. Cy5 fluorescence from FISH-TAMB probes was excited at 640 nm and detected at 665 nm. Three-dimensional projections were generated from z-stacks obtained under a 100X objective. Although the images could not reveal enough details within the cells (Movie S1), the result indicated that 3D live-cell imaging of FISH-TAMB-labeled cells could be possible in the future.

**SI RESULTS & DISCUSSION**

In addition to E. coli *mcr*A^+^ cells, uninduced and induced *E. coli* *lac*Zα^+^ cells labeled with *lac*Zα FISH-TAMB probes were analyzed. More than half of the uninduced *E. coli* *lac*Zα^+^ cells (54.13 ± 7.19%) were assigned as FISH-TAMB-labeled (Table 1), which was significantly more than uninduced *E. coli* *mcr*A^+^ cells being labeled by *mcr*A FISH-TAMB probes. The higher percentage of uninduced *E. coli* *lac*Zα^+^ cells expressing the *lac*Zα gene showed that the *E. coli* *lac*Zα^+^ cells had a higher basal expression level of gene of interest than *E. coli* *mcr*A^+^ cells had. As there were more *E. coli* *lac*Zα^+^ cells detected by FISH-TAMB (74.15 ± 7.04%) after IPTG induction than before IPTG induction, induction did promote the expression of the *lac*Zα gene. The exact reason why nearly one-quarter of the induced cells were not labeled by *lac*Zα FISH-TAMB probes is not clear.

**SUPPLEMENTARY FIGURES**

**Fig. S1. Integrity of *mcr*A MB as a function of temperature.** Melting curve analysis of *mcr*A MB (Cy3 fluorescence) containing 0-5 mM MgCl_2_. (i) Unbound *mcr*A MB. (ii) *mcr*A MB hybridized to target oligonucleotide sequence. (iii) Dissociation of MB from target oligonucleotide sequence. (iv) High temperature results in loss of structural integrity, and random coiling of MB sequence.


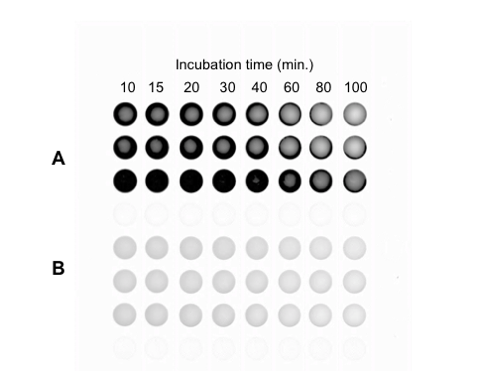


**Fig. S2**. **MB fluorescence lifetime.** An *in vitro* hybridization time series experiment monitored Cy5 fluorescence in **(A)** bound (40 pmol MB + 40 pmol *mcr*A target oligo) and **(B)** 40 pmol unbound MB.


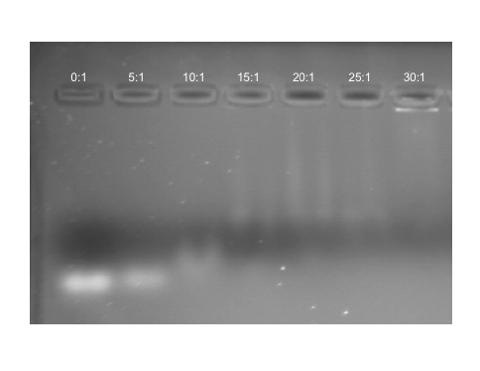


**Fig. S3**. **Complexation between cell-penetrating peptide R9 and *mcr*A MB sequences in fixed R9:MB molar ratios.** Unbound MB shows up as white bands in the gel and complete complexation of all MB to R9 is evidenced by the lack of a physical band. Optimal R9:MB ratio was determined to be 20:1.


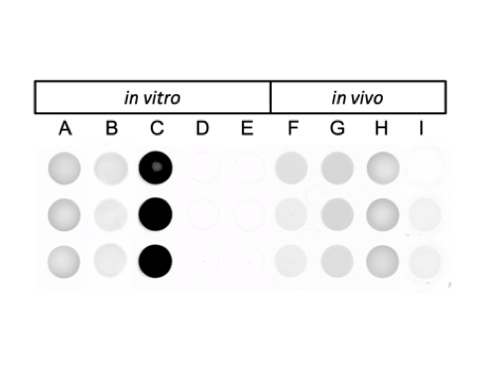


**Fig. S4. Fluorescence of MB and FISH-TAMB probes in the absence (*in vitro*) and presence (*in vivo*) of cells.** Cell-free reactions in 1× DPBS solution include (A) MB-only control (i.e. 40 pmol *mcr*A MBs); (B) MB+non-specific-target control (i.e. 40 pmol *mcr*A MBs and 40 pmol *pmo*A oligonucleotide; (C) MB+specific-target control (i.e. 40 pmol *mcr*A MBs and 40 pmol *mcr*A oligonucleotide); (D) FISH-TAMB-only control (i.e. equivalent to 100 pmol *mcr*A MBs); and (E) FISH-TAMB+specific-target control (i.e. equivalent to 100 pmol *mcr*A MBs and 100 pmol *mcr*A oligonucleotide). Cell-containing reactions containing *mcr*A FISH-TAMB probes (equivalent to 100 pmol *mcr*A MBs) in 1× DPBS solution incubated with (F) *M. barkeri*; (G) ANME enrichment culture; (H) *E. coli mcr*A^+^; and (I) *E. coli* *pmo*A^+^. The grayscale intensity values (mean ± SEM) quantified using ImageJ2 for the different treatments are (A) 211 ± 1.7; (B) 232 ± 2.5; (C) 2 ± 2.4; (D) 253 ± 0.3; (E) 253 ± 0.2; (F) 232 ± 4; (G) 217 ± 2.6; (H) 210 ± 1.6; and (I) 246 ± 4.5.


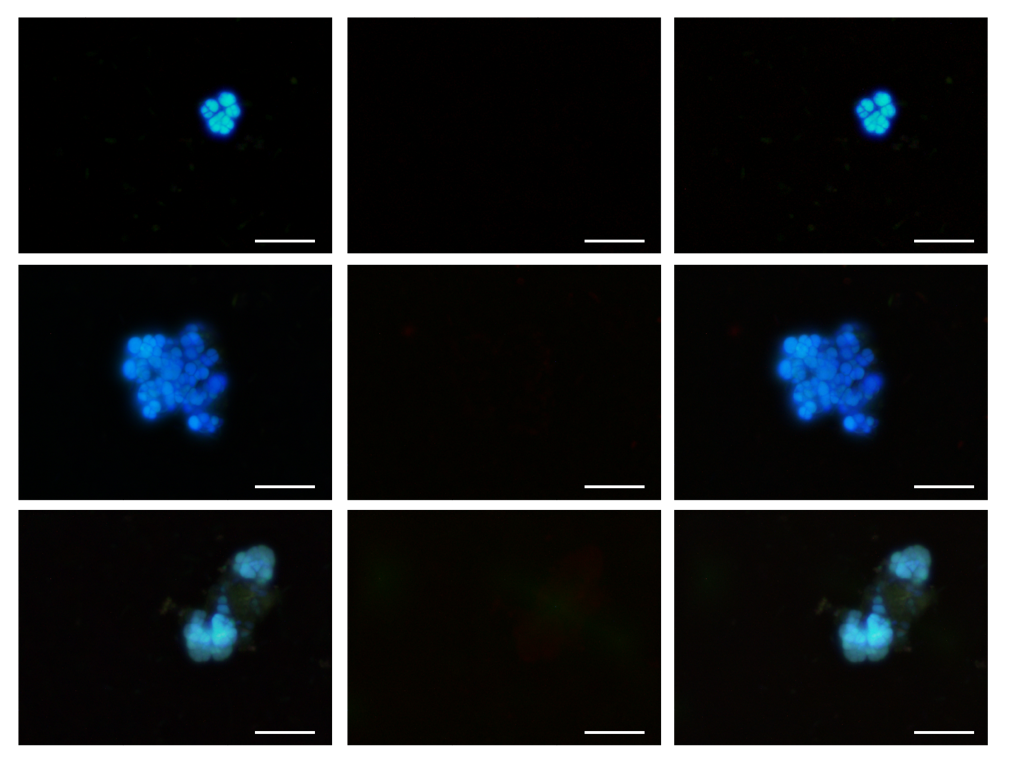


**Fig. S5 Additional micrographs of O_2_-exposed *M. barkeri* cells labeled by *mcr*A FISH-TAMB probes.** Blue channel micrographs are on the left, red channel micrographs are in the middle, and on the right are composite. No Cy5 fluorescence was observed. Scale bar 10 μm.

**Fig. S6 Flow cytometry data of FISH-TAMB targeting *mcr*A mRNA in ANME enrichment.** ANME cells detected as a result of F420 coenzyme autofluorescence are indicated by the population gated in light blue. FISH-TAMB-labeled cells are indicated by the population gated in red and orange for singular and aggregates, respectively. Oxidized F420 coenzyme was excited at 405 mm and emitted fluorescence collected via 450/50 nm bandpass filter (Pacific Blue), whereas Cy5 was excited at 640 nm and emitted fluorescence collected via 670/30 nm bandpass filter. FSC-A stands for forward-scattered density.

**Video S1. A Three-dimensional projection of *E. coli* *lac*Zα^+^ living cells labeled by *lac*Zα FISH-TAMB probes**. The blue areas indicate DAPI-stained cells, whereas the red areas indicate Cy5 fluorescence from the *lac*Zα FISH-TAMB probes. Background noise was attenuated through post-image processing.

**Video S2. *In situ* monitoring of living cells in the ANME enrichment culture.** Video clips (10 frames per second) visualize for 14.5 hours of brightfield and Cy5-fluorescence imaging. Scale bar 5 µm.

**SUPPLEMENTARY TABLES**

**Table S1**: Geochemical data for BE326 BH2 fracture fluid collected 24 June 2016. “< d.l.”, below detection limit.

| Temperature (˚C) | 31.8 |
| --- | --- |
| pH | 8.2 |
| pe | -3.8 |
| Total Dissolved Solid (ppt) | 4.3 |
| Conductivity (mS cm^-1^) | 8.7 |
| H_2_S (ppm) | < 0.1 |
| H_2_O_2_ (ppm) | <0.1 |
| Fe^2+^ (ppm) | 0.1 |
| Total Fe (ppm) | 0.2 |
| PO_4_^3-^ (ppm) | < 0.1 |
| HCOO^-^ (ppm) | < d.l. |
| CH_3_COOH (ppm) | < d.l. |
| NO_2_^-^ (ppm) | 0.52 ± 0.05 |
| NO_3_^-^ (ppm) | 0.01 |
| SO_4_^2-^ (ppt) | 7.43 ± 0.19 |
| Br^-^ (ppm) | 9.14 ± 0.22 |
| O_2_ | 0.2% |
| N_2_ | 5.0% |
| H_2_ | 0.1% |
| CH_4_ | 90.3% |
| CO_2_ | 4.4% |
| He | 0.02% |
| water:gas flow rate ratio | 320:1 |

**Table S2.** Fluorescence intensity of bound and unbound *mcr*A-targeting MB as a function of salinity and temperature.

**REFERENCES**

1. Anderson KL, Apolinario EE, Sowers KR (2012) Desiccation as a long-term survival mechanism for the archaeon *Methanosarcina barkeri*. Appl Environ Microbiol 78:1473–1479. https://doi.org/10.1128/AEM.06964-11

2. Luton PE, Wayne JM, Sharp RJ, Riley PW (2002) The *mcr*A gene as an alternative to 16S rRNA in the phylogenetic analysis of methanogen populations in landfill. Microbiology 148:3521–3530

3. Steinberg LM, Regan JM (2008) Phylogenetic comparison of the methanogenic communities from an acidic, oligotrophic fen and an anaerobic digester treating municipal wastewater sludge. Appl Environ Microbiol 74:6663–6671. https://doi.org/10.1128/AEM.00553-08

4. Lau MCY, Cameron C, Magnabosco C, et al (2014) Phylogeny and phylogeography of functional genes shared among seven terrestrial subsurface metagenomes reveal N-cycling and microbial evolutionary relationships. Front Microbiol 5:531. https://doi.org/10.3389/jmicb.2014.00531

5. Holmes AJ, Costello A, Lidstrom ME, Murrell JC (1995) Evidence that participate methane monooxygenase and ammonia monooxygenase may be evolutionarily related. FEMS Microbiol Lett 132:203–208. https://doi.org/10.1016/0378-1097(95)00311-R

6. Luesken FA, Zhu B, van Alen TA, et al (2011) *pmo*A primers for detection of anaerobic methanotrophs. Appl Environ Microbiol 77:3877–3880. https://doi.org/10.1128/AEM.02960-10

7. Magnabosco C, Tekere M, Lau MCY, et al (2014) Comparisons of the composition and biogeographic distribution of the bacterial communities occupying South African thermal springs with those inhabiting deep subsurface fracture water. Front Microbiol 5:679. https://doi.org/10.3389/fmicb.2014.00679

8. Simkus DN, Slater GF, Sherwood Lollar B, et al (2016) Variations in microbial carbon sources and cycling in the deep continental subsurface. Geochim Cosmochim Acta 173:264–283. https://doi.org/10.1016/j.gca.2015.10.003

9. Widdel F, Bak F (1992) Gram-negative mesophilic sulfate-reducing bacteria. Prokaryotes 2nd ed IV:3352–3378. https://doi.org/10.1007/978-1-4757-2191-1

10. Holler T, Widdel F, Knittel K, et al (2011) Thermophilic anaerobic oxidation of methane by marine microbial consortia. ISME J 5:1946–1956. https://doi.org/10.1038/ismej.2011.77

11. Harris RL, Lau MCY, Cadar A, et al (2018) Draft genome sequence of “ *Candidatus* Bathyarchaeota” archaeon BE326-BA-RLH, an uncultured denitrifier and putative anaerobic methanotroph from South Africa’s deep continental biosphere. Microbiol Resour Announc 7:e01295-18. https://doi.org/10.1128/MRA.01295-18

12. Chen S, Zhou Y, Chen Y, Gu J (2018) fastp: an ultra-fast all-in-one FASTQ preprocessor. Bioinformatics 34:i884–i890. https://doi.org/10.1093/bioinformatics/bty560

13. Bankevich A, Nurk S, Antipov D, et al (2012) SPAdes: a new genome assembly algorithm and its applications to single-cell sequencing. J Comput Biol 19:455–477. https://doi.org/10.1089/cmb.2012.0021

14. Menzel P, Ng KL, Krogh A (2016) Fast and sensitive taxonomic classification for metagenomics with Kaiju. Nat Commun 7:11257. https://doi.org/10.1038/ncomms11257
